# Changes in the Constitution of the American Medical Association

**Published:** 1887-07

**Authors:** 


### CHANGES IN THE CONSTITUTION OF THE AMERICAN MEDICAL ASSOCIATION.

The report of the Committee appointed at the St. Louis meeting under the following resolution excited much debate:

*Resolved*, That a committee of nine members, including the President, the President-elect and the four Vice-Presidents-elect, be appointed by the chair to consider the various propositions looking to the amendment of the organic law of the association by the *establishment of branches*, or in any other way; said committee to report at the next regular annual meeting what measures of organization, if any, may be desirable.

The report was quite lengthy and did not recommend any radical changes in the organic law of the association.

It is recommended that the amendment to section II, relating to "members by application," be so amended as to read:

*Members* by *Application* shall consist of such members of the State, County and District Medical Societies entitled to representation in this Association, as shall make application in writing to the Treasurer, and accompany said application with a certificate of good standing signed by the President and Secretary of the society of which they are members, and the amount of the annual membership fee, $5. They shall have their names upon the roll and have all the rights and privileges accorded to *Permanent Members*, and shall retain their membership on the same terms.

This we consider an exceedingly wise amendment, allowing, as it does, members of any recognized state or local society to become permanent members without attendance upon an annual meeting.

The committee recommended that, from the *fifth section* of the *Constitution*, relating to "*Standing Committees*," the *first* and *third paragraphs* should be stricken out, leaving intact only the second paragraph, relating to the "Committee of Arrangements." In place of the first paragraph they advised the insertion of the following important provision, viz.:

The General Committee or Council shall be composed of *two members* from each State and Territorial Medical Society entitled to representation by delegates in the Association, and from the Medical Departments of the U. S. Army, Navy and Marine Hospital Service. They shall be chosen by the members registered and present at each annual meeting, from each State, Territory, and from the Medical Corps of the U. S. Army, Navy and Marine Hospital Service, acting separately, on the third day of each annual meeting; each delegation reporting the names of the members chosen to the Permanent Secretary of the Association on the same day, that they may be announced by him at the opening of the morning session of the fourth day. At the first election each delegation shall choose *two* members of the General Committee, one of whom shall serve *one* year and the other *two* years, and at each annual election thereafter one member shall be chosen to serve for two years, thus making the term of office of members of the General Committee *two* years. It shall be the duty of the General Committee, thus constituted, to organize by choosing annually a Chairman and Secretary, and such sub-committees as may be found necessary to facilitate the work that may be assigned to it; to meet annually at the place and on the day preceding each annual meeting of this Association, and as often during that week as may be necessary; to nominate, on the third day of each annual meeting, all the general officers of the Association (none of whom shall be members of its own body), the members of the Committee of Arrangements, the Committee on Necrology, seven members of the Judicial Council, and three members of the Board of Trustees for Publication for election by the Association; to recommend the place and time for holding the next annual meeting; and to consider and report upon all subjects that may be referred to it by vote of the Association. The presence of one-third of the whole number of members elected to the General Committee shall constitute a quorum for the transaction of business. If, at any annual meeting of the Association, it shall be found at the close of the general meeting of the first day that a quorum of the General Committee is not present, it shall be the duty of the President and Permanent Secretary to fill the vacancies in the Committee temporarily by selections from the lists of delegates registered as present from the States to which the vacancies belong.

We confess that the wording of this section is to us very ambiguous. According to our interpretation it has some very objectionable features. We believe the object of the committee was to avoid wire pulling or manipulating the committee entrusted with nominating officers. If the proposed amendment is adopted at Cincinnati it will furnish the best opportunity that could be desired to further personal ambitions. If it were possible to influence a committee in a few hours, what will not be possible in a whole year or two? We think this committee should have general supervision of the business affairs of the association, but the committee to whom is entrusted the nomination of officers should be selected on the morning of the third day of each annual meeting and report on the morning of the last day. This would reduce to a minimum the chances for any individual to further personal or other ambitions. It must be admitted that a medical association is human, and that if by any honorable means the methods of the better class of politicians can be carried out they will most assuredly be prosecuted with a vigor that might surprise the veteran statesman. Every possible measure should be guarded against which will permit, as one member well said, of all the business of the association being transacted by members on the stage.

The committee recommends the adoption of the following provision and its incorporation in the Constitution, as a substitute for the third paragraph of section five relating to the "Committee of Publication," viz.:

The *Board of Trustees* shall consist of nine members, three of whom shall be elected annually on the nomination of the standing General Committee, and shall serve for three years. It shall be the duty of this Board to provide for and superintend the publication and distribution of all such proceedings, transactions, and memoirs of the Association as may be ordered to be published, and in such manner as the Association may direct; and in doing this, it shall have authority to appoint an editor and such assistants, and determine their salaries, and procure and control such materials as may be necessary for the accomplishment of the work assigned to it. To further facilitate its work, it shall be the duty of the Secretaries of the Association and of the several Sections, during each annual meeting, or as soon thereafter as practicable, to deliver to the Board, or such editor or agent as it shall appoint, all such records of proceedings, reports, addresses, papers, and other documents as may have been ordered for publication, either in the general sessions or in the Sections. All moneys received by the Board of Trustees or its agents, resulting from the discharge of the duties assigned them, must be paid to the Treasurer of the Association, and all orders on the Treasurer for disbursements of money in any way connected with the work of publication, must be endorsed by the President of the Board of Trustees. It shall be the further duty of the said Board of Trustees to hold the official bond of the Treasurer for the faithful execution of his office; to annually audit and authenticate his accounts, and present a statement of the same in its annual report to the Association; which report shall also specify the character and cost of all the publications for the Association during the year, the number of copies still on hand, and the amount of all other property belonging to the Association under its control, with such suggestions as it may deem necessary.

The wisdom of adopting this provision, in its entirety, we doubt. It will be noticed that the Board of Trustees are required to turn over all moneys into the hands of the Treasurer; that all orders on the Treasurer for the disbursement of funds must be endorsed by the President of the Board of Trustees; and farther, that the Board of Trustees holds the official bond of the Treasurer and audits his accounts. This plan, if adopted, will make a very pretty mutual admiration society. The Board of Trustees incurs nine-tenths of the whole expense of the Association, its orders are paid by the Treasurer, and then the Board audits its own accounts. How could it find a mistake or any evidence of extravagance under such a complete system of oversight? The business management of the Association has been too long at fault. In these times it is as necessary that the affairs of a medical association should be conducted according to business principles as that a banking institution is held to strict accountability for its acts. The proposed changes in the constitution will be voted on at the next meeting in Cincinnati. They should be most carefully considered by every member of the profession that the action there taken may be for the best interests of the association as a whole.

